# An Area-Efficient Readout Circuit for a High-SNR Triple-Gain LOFIC CMOS Image Sensor †

**DOI:** 10.3390/s25196093

**Published:** 2025-10-02

**Authors:** Ai Otani, Hiroaki Ogawa, Ken Miyauchi, Yuki Morikawa, Hideki Owada, Isao Takayanagi, Shunsuke Okura

**Affiliations:** 1 Research Organization of Science and Engineering, Ritsumeikan University, 1-1-1 Noji-Higashi, Kusatsu 525-8577, Japan; ri0096xe@ed.ritsumei.ac.jp (A.O.); 21v00531@gst.ritsumei.ac.jp (H.O.);; 2 Brillnics Japan Inc., Omori Prime Building 7F, 6-21-12 Minami-Oi, Shinagawa-ku, Tokyo 140-0013, Japan; morikawa.yuki@brillnics.com (Y.M.); owada.hideki@brillnics.com (H.O.); takayanagi.isao@brillnics.com (I.T.)

**Keywords:** CMOS image sensor, LOFIC, HDR, readout circuit, high SNR

## Abstract

A lateral overflow integration capacitor (LOFIC) CMOS image sensor (CIS) can achieve high-dynamic-range (HDR) imaging by combining a low-conversion-gain (LCG) signal with a high-conversion-gain (HCG) signal. However, the signal-to-noise ratio (SNR) drops at the switching point from HCG signal to LCG signal due to the significant pixel noise in the LCG signal. To address this issue, a triple-gain LOFIC CIS with a middle-conversion-gain (MCG) signal has been introduced. In this work, we propose an area-efficient readout circuit for the triple-gain LOFIC CIS, using amplifier and capacitor sharing techniques to process the HCG, MCG, and LCG signals. A test chip of the proposed readout circuit was fabricated using the 0.18μm CMOS process. The area overhead was only 7.6%, and the SNR drop was improved by 8.05 dB compared to the readout circuit for a dual-gain LOFIC CIS.

## 1. Introduction

CMOS image sensors (CISs) used in conditions of extreme illumination (e.g., outside) require high-dynamic-range (HDR) imaging technology to avoid underexposure and overexposure of various objects in a scene. Many approaches have been proposed to realize HDR CISs, such as logarithmic compression [1,2], multiple-exposure HDR (MEHDR) [3,4,5,6], dual-conversion-gain (DCG) pixels [7,8,9,10,11,12], and lateral overflow integration capacitor (LOFIC) pixels [7,13,14,15,16,17,18,19,20,21]. LOFIC pixels promise a linear response and reduced motion artifacts. MEHDR images that combine two images taken at different exposure times will cause motion artifacts due to the misalignment of the timings. On the other hand, SEHDR systems, such as LOFIC, combine two images taken at the same exposure time with different gains: low conversion gain (LCG) and high conversion gain (HCG). Thus, SEHDR has an advantage for mitigating motion artifacts [22]. The LOFIC pixel uses an LCG signal to process large maximum signal charges and an HCG signal to reduce dark noise for HDR imaging. However, the LCG pixel reset noise is not canceled due to uncorrelated double sampling, while the HCG pixel reset noise is canceled by correlated double sampling (CDS). Consequently, the signal-to-noise ratio (SNR) drops at the switching point from an HCG to LCG signal. The SNR drop can be mitigated by adding a middle-conversion-gain (MCG) signal, which shifts the switching point of the LCG signal towards a higher signal level. Although several architectures using MCG signals [19,20,21,23] have been proposed, these studies have primarily focused on increasing the dynamic range by enlarging the pixel size or reducing motion artifacts. Additionally, the peripheral circuit area is large because a three-channel readout circuit is required to read the HCG, MCG, and LCG signals [24]. In this work, we propose an area-efficient readout circuit for the triple-gain LOFIC CIS by using amplifier and capacitor sharing techniques based on our previous design for a conventional dual-gain LOFIC CIS [25]. The proposed readout circuit is designed to minimize the SNR drop while maintaining a small pixel area and suppressing motion artifacts. A test chip of the readout circuit was fabricated using a 0.18μm CMOS process and was evaluated to estimate the SNR. Our contributions are summarized as follows:

- To mitigate the SNR drop, we propose the triple-gain LOFIC CIS without enlarging the pixel area or degrading motion artifacts.
- We propose a readout circuit that is area-efficient for the triple-gain LOFIC CIS.
- We evaluate a fabricated test chip and estimate the SNR.

Section 2 provides a voltage-level diagram for designing a triple-gain readout circuit for an LOFIC pixel. The proposed readout circuit is described in Section 3, followed by the measurement results of a fabricated test chip shown in Section 4. Section 5 provides a summary of this work.

### Updates from the Conference Proceeding

**Figure 1:**
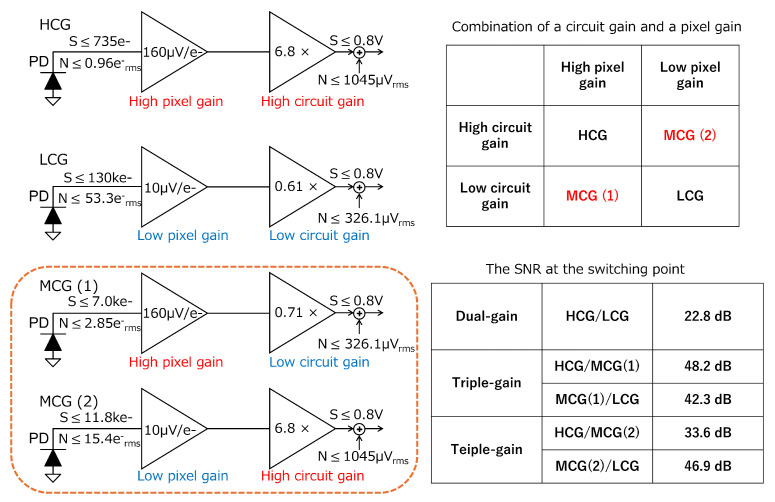
Concept of processing HCG, LCG, and MCG signals.

A conference proceeding version of this paper appeared in the 2025 IEEE International Symposium on Circuits and Systems (ISCAS) [26], and this extended version contains the following new content:

- Pixel- and circuit-gain combination for the MCG signal (Section 2.1, Figure 1).
- Details of the baseline circuit and comparison with the proposed readout circuit (Section 3.1, Figure 3).
- Noise analysis of baseline circuit and proposed readout circuit (Figure 4 and Figure 6).
- A discussion of this study and a comparison with other works (Section 4).

## 2. Concept-Level Design

This section presents a method to generate the MCG signal, in addition to the conventional HCG and LCG signals, without increasing the pixel area. The MCG signal can be obtained either by combining a high pixel gain with a low circuit gain or a low pixel gain with a high circuit gain. First, the combination for the MCG signal is determined based on the circuit noise performance. Then, a voltage-level diagram is examined for the triple-gain LOFIC CIS using the selected MCG configuration.

### 2.1. Pixel- and Circuit-Gain Combination for the MCG Signal

In our conventional dual-gain LOFIC CIS [25], the HCG signal is generated by a high pixel gain and a high circuit gain, while the LCG signal is generated by a low pixel gain and a low circuit gain, as shown in Figure 1. The full well capacity (FWC) for HCG signal is $735\;e^{-}$, provided by $S_{max}$/$A_{cir}$/$A_{pix}$, where $S_{max}$ is a maximum output signal of 0.8 V; $A_{cir}$ and $A_{pix}$ are circuit gain, including a pixel source-follower (SF) gain of 6.8× and a pixel conversion gain of $160\;\mu V/e^{-}$ [16], respectively. The floor noise for HCG signal is $0.96\;e_{\mathrm{rms}}^{-}$, provided by *N*/$A_{cir}$/$A_{pix}$, where *N* is the output floor noise of $1045\;\mu V_{\mathrm{rms}}$ based on previous work [25]. While the input-referred noise is very small, the FWC is limited. Similarly, the FWC for LCG signal is $130\;{\mathrm{ke}}^{-}$, provided by $S_{max}$/$A_{cir}$/$A_{pix}$, where $S_{max}$ is a maximum output signal of 0.8 V; $A_{cir}$ and $A_{pix}$ are circuit gain, including a pixel SF gain of 0.61× and a pixel conversion gain of $10\;\mu V/e^{-}$ [16], respectively. The floor noise for LCG signal is $53.3\;e_{\mathrm{rms}}^{-}$, provided by *N*/$A_{cir}$/$A_{pix}$, where *N* is the output floor noise of $326.1\;\mu V_{\mathrm{rms}}$ based on previous work [25]. While the FWC is very large, the input-referred noise is considerably large. Thus, the signal-to-noise ratio (SNR) at the switching point from the HCG to LCG signal is provided by $735\;e^{-}/53.3\;e_{\mathrm{rms}}^{-}=22.8$ dB, where the noise will be visible because the SNR is below 30 dB [27].

To generate the MCG signal, two candidates are considered: (1) high pixel gain combined with low circuit gain; and (2) low pixel gain combined with high circuit gain. This is because the selectable pixel gain is constrained to either high or low in order to maintain a compact pixel area, as in the conventional dual-gain LOFIC pixels [16]. The FWC for MCG (1) signal is $7.0\;{\mathrm{ke}}^{-}$, provided by $S_{max}$/$A_{cir}$/$A_{pix}$, where $S_{max}$ is a maximum output signal of 0.8 V; $A_{cir}$ and $A_{pix}$ are circuit gain, including a pixel SF of 0.71× and a pixel conversion gain of $160\;\mu V/e^{-}$ [16], respectively. The floor noise for MCG (1) signal is $2.85\;e_{\mathrm{rms}}^{-}$, provided by *N*/$A_{cir}$/$A_{pix}$, where *N* is the output floor noise of $326.1\;\mu V_{\mathrm{rms}}$ based on previous work [25]. The SNR at the switching point from HCG to MCG (1) is $735\;e^{-}/2.85\;e_{\mathrm{rms}}^{-}=48.2$ dB. On the other hand, the SNR at the switching point from MCG (1) to LCG is $7.0\;{\mathrm{ke}}^{-}/53.3\;e_{\mathrm{rms}}^{-}=42.3$ dB. Similarly, the input FWC values of MCG (2) are $11.8\;{\mathrm{ke}}^{-}$, provided by $S_{max}$/$A_{cir}$/$A_{pix}$, where $S_{max}$ is a maximum output signal of 0.8 V; $A_{cir}$ and $A_{pix}$ are circuit gain, including pixel SF of 6.8× and a pixel conversion gain of $10\;\mu V/e^{-}$ [16], respectively. The floor noise for MCG (2) signal is $15.4\;e_{\mathrm{rms}}^{-}$, provided by *N*/$A_{cir}$/$A_{pix}$, where *N* is the output floor noise of $1045\;\mu V_{\mathrm{rms}}$ based on previous work [25]. The SNR at the switching point from HCG to MCG (2) is $735\;e^{-}/15.4\;e_{\mathrm{rms}}^{-}=33.6$ dB. On the other hand, the SNR at the switching point from MCG (2) to LCG is $11.8\;{\mathrm{ke}}^{-}/53.3\;e_{\mathrm{rms}}^{-}=46.9$ dB. Based on these rough estimations, MCG (1) is selected because of its larger margin relative to the 30 dB criterion. Additionally, it is noted that photon shot noise dominates at the switching point to the LCG signal.

### 2.2. Voltage-Level Diagram

**Figure 2:**
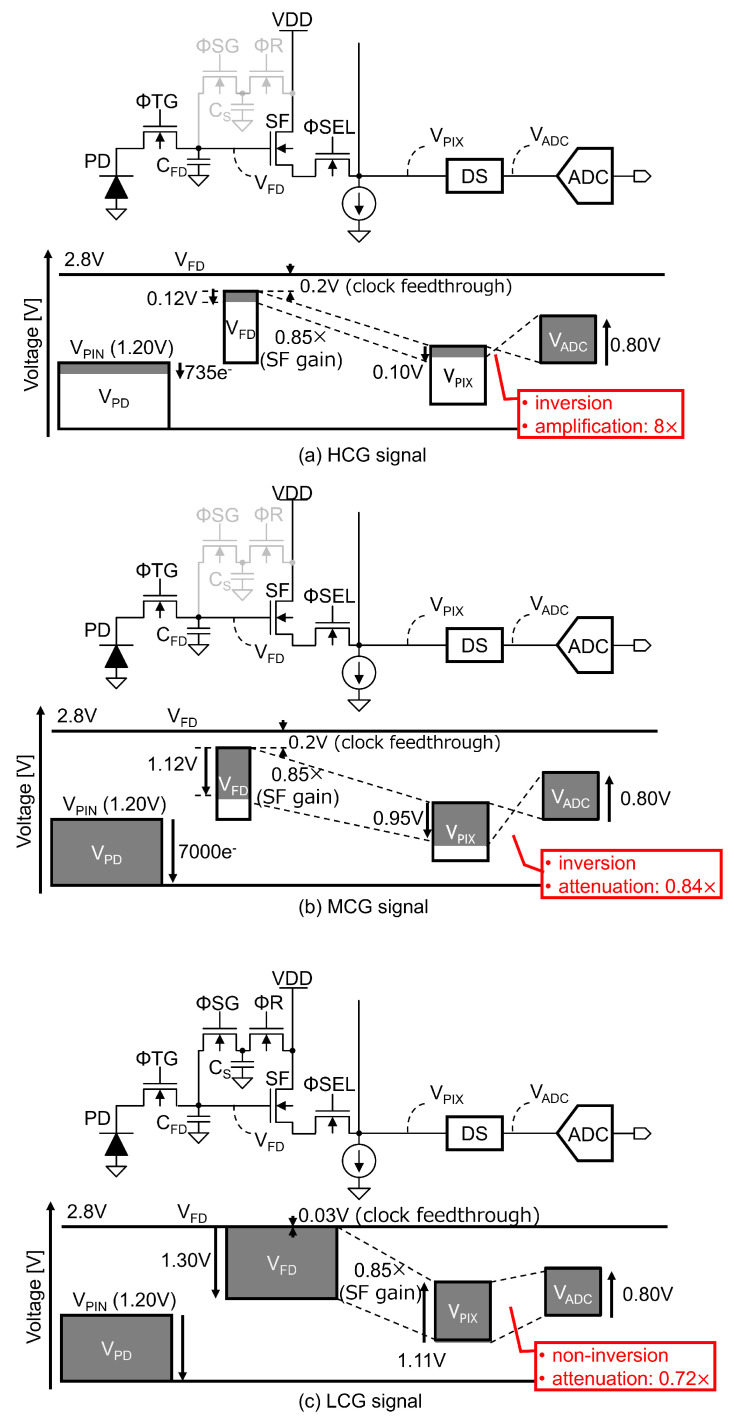
The level diagram of LOFIC CIS: (**a**) HCG signal; (**b**) MCG signal; (**c**) LCG signal.

For the triple-gain LOFIC CIS employing the selected MCG configuration, combining the high pixel gain with the low circuit gain, a detailed voltage-level diagram was designed, as shown in Figure 2, including the pinning voltage of the photo-diode (PD) ($V_{PIN}$), the signal voltage swing at the floating diffusion (FD) node ($V_{FD}$) and at the pixel output ($V_{PIX}$), and the input to an ADC ($V_{ADC}$). For the HCG signal shown in Figure 2a, the voltage gain of the double-sampling (DS) circuit is selected as 8×, such that the input-referred circuit noise can be decreased and a high dynamic range can be achieved. The input window of the DS circuit, which derives from the difference between the reset level and the signal level from the pixel output, is set to 0.10 V for the 0.80 V ADC input window [28], and the signal voltage swing at the FD node is limited to below 0.12 V with a 0.85× SF gain. For the MCG signal shown in Figure 2b, the PD FWC and $C_{FD}$ are assumed to be $7000\;e^{-}$ [16] and 1 fF, respectively, and the maximum signal voltage swing at the FD node is 1.12 V. Even though the clock feed-through on a small $C_{FD}$ can be as large as 0.20 V when the ΦSG is turned off, there is still a sufficient voltage margin to transfer the photo-electrons integrated in the PD to the FD node. The input window of the DS circuit is 0.95 V with an SF, and the voltage gain of the DS circuit is set to 0.84× for the ADC input window. For the LCG signal shown in Figure 2c, the maximum voltage swing at the FD node is set to 1.30 V in order to read the photo-electrons integrated in the PD, FD, and $C_{S}$. The input window of the DS circuit is set to 1.11 V with an SF, and the gain of the DS circuit is set to 0.72× for the ADC input window. The clock feed-through on $C_{FD}$ and $C_{S}$ is as small as 0.03 V when the ΦR is turned off. Hence, the voltage margin required to transfer the photo-electrons integrated in the PD to the FD node remains even for large-FD voltage swings. These level diagrams show that the HCG and MCG signals are inverted, while the LCG signal is not, which enables sharing an ADC for the triple-gain signals.

## 3. Proposed Readout Circuit for a Triple-Gain LOFIC CIS

As the three-channel readout circuit [24] used to read HCG, MCG, and LCG signals in triple-gain LOFIC CIS leads to an increased chip size, we propose a single-channel readout circuit for the triple-gain LOFIC CIS. First, a baseline circuit is presented, followed by an enhanced design aimed at reducing circuit noise.

### 3.1. Baseline Readout Circuit

**Figure 3:**
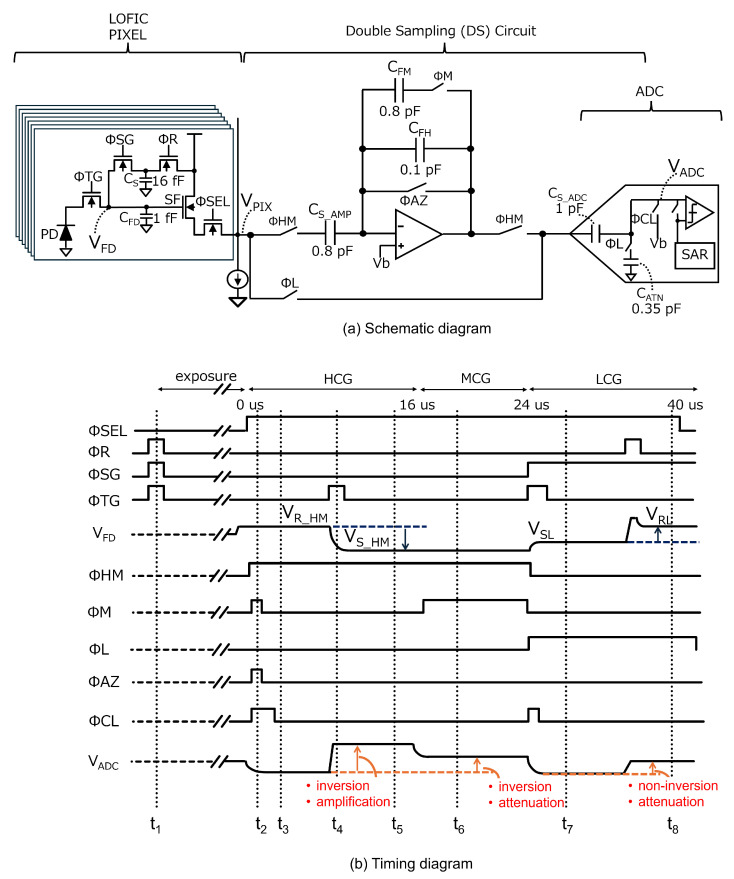
(**a**) Schematic diagram and (**b**) timing diagram of the baseline readout circuit.

**Figure 4:**
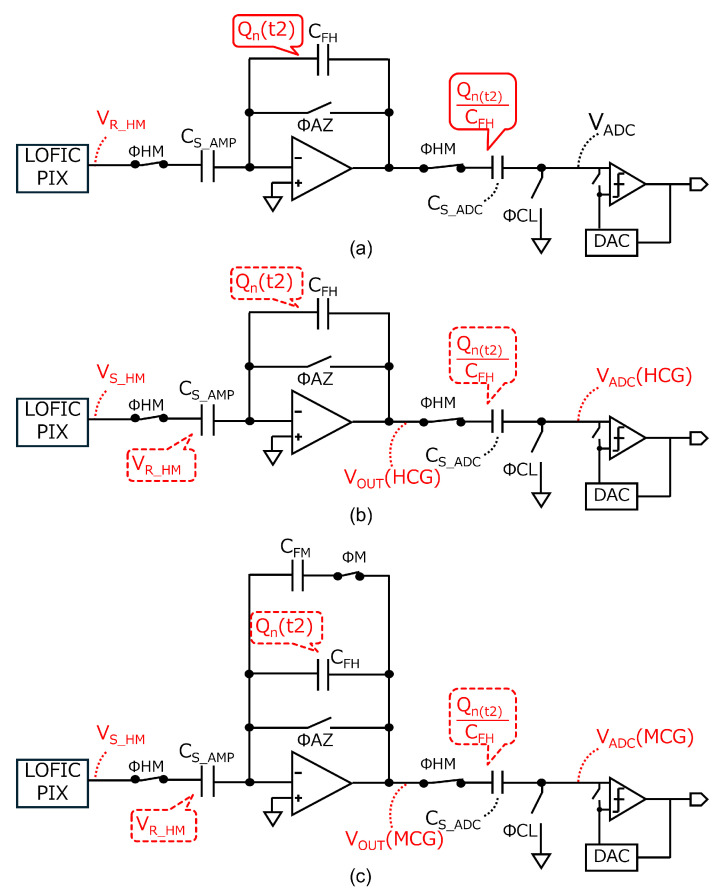
Equivalent circuits of the baseline circuit (**a**) at $t_{3}$, (**b**) at $t_{5}$, and (**c**) at $t_{6}$ to analyze the HCG and MCG outputs.

A baseline readout circuit for the triple-gain LOFIC CIS is shown in Figure 3. An amplifier and capacitor are shared for the HCG and MCG signals, and an attenuation capacitor for the LCG signal is shared with a sampling capacitor of an ADC, in which the ADC subsequently processes the HCG, MCG, and LCG signals. This baseline readout circuit is based on our previous design for a conventional dual-gain LOFIC CIS [25], with only one additional feedback capacitor $C_{FM}$ [29,30] introduced to implement the inverted attenuator for the MCG signal. Figure 3b shows a timing diagram of the baseline readout circuit. The total conversion period is 40μs and has increased by 8μs compared to dual-gain LOFIC CIS [25] but remains less than 1.5 times. First, ΦSG, ΦR, and ΦTG are all toggled to reset the PD, the floating diffusion capacitor $C_{FD}$, and an overflow photo-electron integration capacitor $C_{S}$ at $t_{1}$ prior to starting the exposure. When the exposure is complete, ΦSEL becomes high in a given pixel row, and the pixel reset levels for HCG and MCG ($V_{R\_HM}$) are output from the selected pixel at $t_{2}$. At this time, ΦHM values are high to store $A_{SF}\cdot \left(V_{R\_HM}-V_{GS\_SF}\right)$ in $C_{S\_AMP}$, where $A_{SF}$ and $V_{GS\_SF}$ are the pixel SF gain and the offset of the pixel SF transistor, respectively. The amplifier reset noise, $Q_{n}\left(t2\right)$, is generated on the virtual ground node, and $Q_{n}\left(t2\right)/C_{FH}$ is sampled in $C_{S\_ADC}$ after ΦAZ is turned off at $t_{3}$. Figure 4a shows the equivalent circuits at $t_{3}$, where the bias voltage Vb and noise other than the amplifier reset noise are considered to be 0 for simplicity.

By toggling ΦTG at $t_{4}$, the pixel signal levels for HCG and MCG ($V_{S\_HM}$), provided by $A_{SF}\cdot \left(V_{R\_HM}-Q_{SIG}/C_{FD}-V_{GS\_SF}\right)$, where $Q_{SIG}$ is the signal charge transferred from the PD, are output from the pixel at $t_{5}$. Figure 4b shows the equivalent circuits at $t_{5}$. At this time, the output signal from the amplifier, $V_{OUT}\left(HCG\right)$, is inverted and amplified using $C_{S\_AMP}$ and $C_{FH}$, as provided by

(1) $$V_{OUT}\left(HCG\right)=A_{SF}\cdot \frac{C_{S\_AMP}}{C_{FH}}\cdot \frac{Q_{SIG}}{C_{FD}}+\frac{Q_{n}\left(t2\right)}{C_{FH}}.$$

The HCG signal input to the ADC, $V_{ADC}\left(HCG\right)$, is thus provided by

(2) $$\begin{array}{cc} V_{ADC}\left(HCG\right) & =V_{OUT}\left(HCG\right)-\frac{Q_{n}\left(t2\right)}{C_{FH}} \end{array}$$

(3) $$\begin{array}{cc} \; & \;\;\;=A_{SF}\cdot \frac{C_{S\_AMP}}{C_{FH}}\cdot \frac{Q_{SIG}}{C_{FD}}, \end{array}$$

where the amplifier reset noise $Q_{n}\left(t2\right)/C_{FH}$ is removed. Subtracting the pixel reset level from the pixel signal level also cancels the pixel reset noise and the offset of the pixel SF transistor $V_{GS\_SF}$. After ΦM becomes high, where Figure 4c is the equivalent circuit, the output signal from amplifier, $V_{OUT}\left(MCG\right)$, is provided by

(4) $$V_{OUT}\left(MCG\right)=A_{SF}\cdot \frac{C_{S\_AMP}}{C_{FH}+C_{FM}}\cdot \frac{Q_{SIG}}{C_{FD}}+\frac{Q_{n}\left(t2\right)}{C_{FH}+C_{FM}},$$

at $t_{6}$. The MCG signal input to the ADC, $V_{ADC}\left(MCG\right)$, is inverted and attenuated, as provided by

(5) $$\begin{array}{cc} V_{ADC}\left(MCG\right) & =V_{OUT}\left(MCG\right)-\frac{Q_{n}\left(t2\right)}{C_{FH}} \end{array}$$

(6) $$\begin{array}{cc} \; & =A_{SF}\cdot \frac{C_{S\_AMP}}{C_{FH}+C_{FM}}\cdot \frac{Q_{SIG}}{C_{FD}}+\frac{Q_{n}\left(t2\right)}{C_{FH}+C_{FM}}-\frac{Q_{n}\left(t2\right)}{C_{FH}}. \end{array}$$

As provided by Equation (6), the amplifier reset noise for the MCG signal is not canceled, which decreases its SNR, resulting in an SNR drop from HCG to MCG. After ΦL and ΦSG become high, the pixel signal level for LCG ($V_{SL}$), provided by $A_{SF}\cdot \left(V_{RL}-\frac{Q_{SIG}}{C_{FD}+C_{S}}-V_{GS\_SF}\right)$, is directly stored in $C_{S\_ADC}$ at $t_{7}$. After ΦR is toggled to reset $C_{FD}$ and $C_{S}$, the pixel reset level for LCG, provided by $A_{SF}\cdot \left(V_{RL}^{\prime }-V_{GS\_SF}\right)$, is output from the pixel array. The non-inverted and attenuated LCG signal, using $C_{S\_ADC}$ and $C_{ATN}$, is input to the ADC at $t_{8}$, as provided by

(7) $$\begin{array}{cc} \; & V_{ADC}\left(LCG\right) \end{array}$$

(8) $$\begin{array}{cc} \; & =A_{SF}\cdot \frac{C_{S\_ADC}}{C_{S\_ADC}+C_{ATN}}\cdot \left(\frac{Q_{SIG}}{C_{FD}+C_{S}}+n_{PIX}\right). \end{array}$$

The voltage difference between $V_{RL}$ and $V_{RL}^{\prime }$ results in high pixel reset noise $n_{PIX}$, provided by $\sqrt{\frac{2kT}{C_{FD}+C_{S}}}$. The pixel reset noise $n_{\mathrm{PIX}}$ is estimated to be $867\;\mu V_{\mathrm{rms}}$, assuming $C_{FD}=1.0\;\mathrm{fF}$ and $C_{S}=16\;\mathrm{fF}$ [16]. LCG pixel reset noise $n_{PIX}$ is not canceled. The SNR of LCG signal is high since LCG has a large signal electron, even if the pixel reset noise $n_{PIX}$ is not canceled. However, the impact of the pixel reset noise $n_{PIX}$ is significant with a small signal electron, leading to the SNR drop. By adding MCG signal, the switching point of the LCG signal shifts towards a higher signal level, resulting in suppression of the SNR drop.

### 3.2. Proposed Circuit

**Figure 5:**
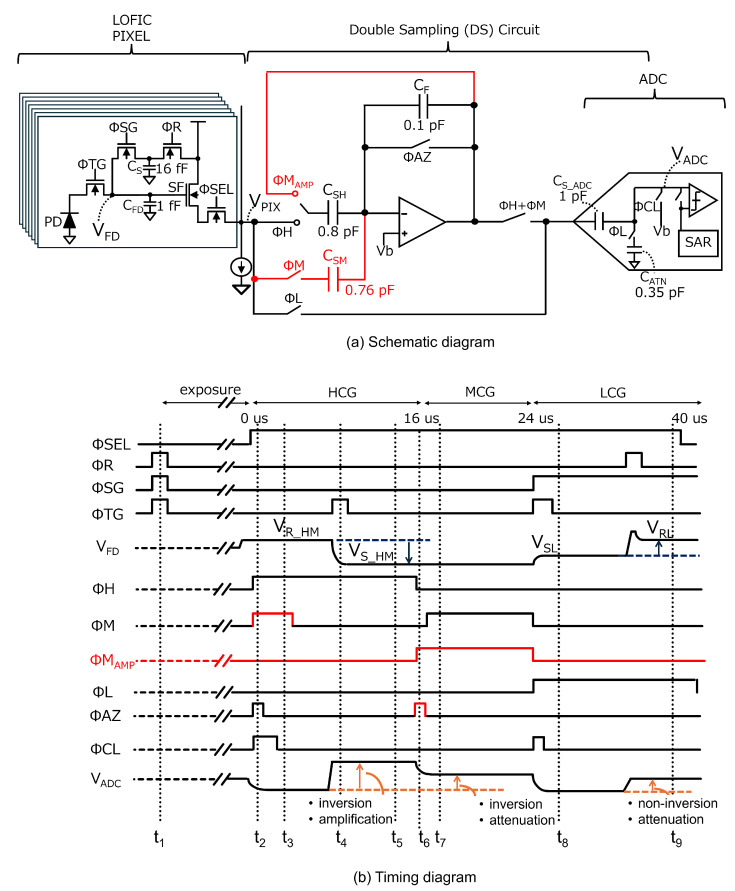
(**a**) Schematic diagram and (**b**) timing diagram of the proposed readout circuit.

**Figure 6:**
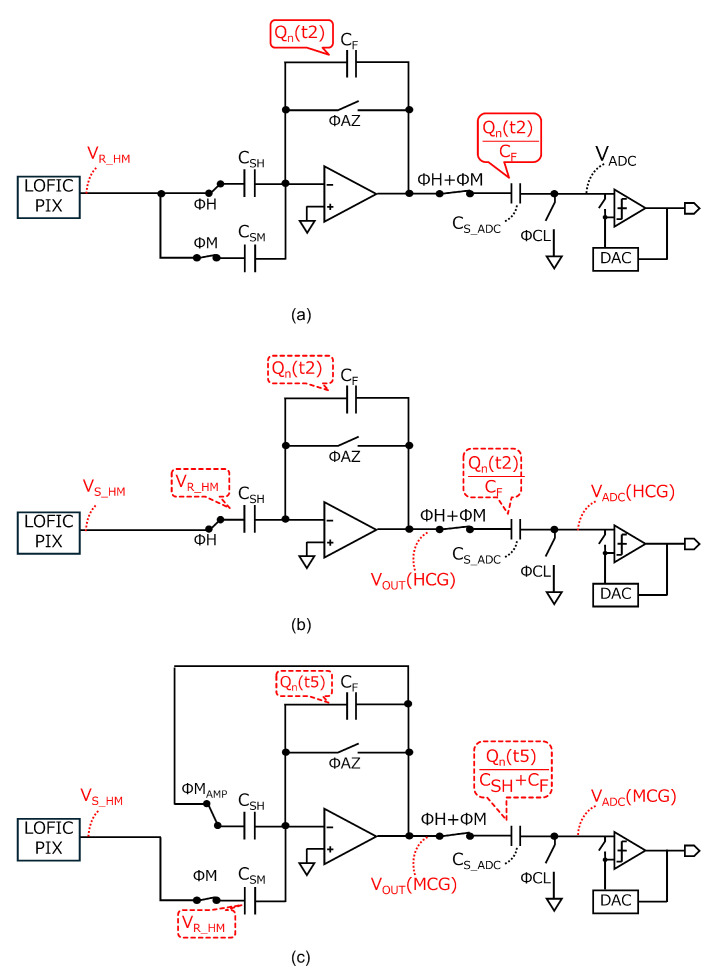
Equivalent circuits of the proposed readout circuit (**a**) at $t_{3}$, (**b**) at $t_{5}$, and (**c**) at $t_{7}$ to analyze the HCG and MCG outputs.

Although the baseline circuit is area-efficient, it does not cancel the amplifier reset noise for the MCG signal, as provided by Equation (6). This causes an SNR drop from HCG to MCG. To solve this problem, we propose a readout circuit, as shown in Figure 5a, consisting of the same capacitance as the baseline circuit. The changes from the baseline circuit are indicated by red lines. Figure 5b shows a timing diagram of the proposed readout circuit. At $t_{2}$, ΦH and ΦM are high, and $A_{SF}\cdot \left(V_{R\_HM}-V_{GS\_SF}\right)$ is stored in $C_{SH}$ and $C_{SM}$, respectively. The amplifier reset noise, $Q_{n}\left(t2\right)/C_{F}$, is sampled in $C_{S\_ADC}$ after ΦAZ is turned off at $t_{3}$. Figure 6a shows the equivalent circuits at $t_{3}$, where Vb and noise other than the amplifier reset noise are considered to be 0 for simplicity.

After ΦM becomes low and $Q_{SIG}$ is transferred from the PD at $t_{4}$, the pixel signal levels for HCG and MCG ($V_{S\_HM}$) are output from the pixel at $t_{5}$, as shown in Figure 6b. At this time, the output signal from amplifier, $V_{OUT}\left(HCG\right)$, is inverted and amplified using $C_{SH}$ and $C_{F}$, as provided by

(9) $$V_{OUT}\left(HCG\right)=A_{SF}\cdot \frac{C_{SH}}{C_{F}}\cdot \frac{Q_{SIG}}{C_{FD}}+\frac{Q_{n}\left(t2\right)}{C_{F}}.$$

The HCG signal input to the ADC, $V_{ADC}\left(HCG\right)$, is thus provided by

(10) $$\begin{array}{cc} V_{ADC}\left(HCG\right)\; & =V_{OUT}\left(HCG\right)-\frac{Q_{n}\left(t2\right)}{C_{F}} \end{array}$$

(11) $$\begin{array}{cc} \; & \;\;\;=A_{SF}\cdot \frac{C_{SH}}{C_{F}}\cdot \frac{Q_{SIG}}{C_{FD}}. \end{array}$$

The amplifier reset noise is canceled for the HCG signal, as in the baseline circuit. Before reading out the MCG signal, $\Phi M_{AMP}$ becomes high, and ΦAZ is toggled to reset the charge stored in $C_{SH}$ and $C_{F}$ at $t_{6}$. At this time, the amplifier reset noise $Q_{n}\left(t_{6}\right)$ is generated, and $Q_{n}\left(t_{6}\right)/\left(C_{SH}+C_{F}\right)$ is sampled in $C_{S\_ADC}$ after ΦAZ is turned off at $t_{7}$. At this time, ΦM is high to set $C_{SM}$ as a sampling capacitor and to reuse $C_{SH}$ as a feedback capacitor, respectively. The equivalent circuit at $t_{7}$ is shown in Figure 6c. The output signal from amplifier, $V_{OUT}\left(MCG\right)$, is inverted and attenuated using $C_{SM}$ and $C_{SH}+C_{F}$, as provided by

(12) $$V_{OUT}\left(MCG\right)=\frac{C_{SM}}{C_{SH}+C_{F}}\cdot A_{SF}\cdot \frac{Q_{SIG}}{C_{FD}}+\frac{Q_{n}\left(t_{6}\right)}{C_{SH}+C_{F}}.$$

The MCG signal input to the ADC, $V_{ADC}\left(MCG\right)$, is thus provided by

(13) $$\begin{array}{cc} V_{ADC}\left(MCG\right) & =V_{OUT}\left(MCG\right)-\frac{Q_{n}\left(t6\right)}{C_{SH}+C_{F}} \end{array}$$

(14) $$\begin{array}{cc} \; & \;\;\;=\frac{C_{SM}}{C_{SH}+C_{F}}\cdot A_{SF}\cdot \frac{Q_{SIG}}{C_{FD}}. \end{array}$$

Due to autozeroing before readout of the MCG signal, the amplifier reset noise is also canceled for the MCG signal. The operation from $t_{8}$ to $t_{9}$ is the same as that described for the baseline circuit from $t_{7}$ to $t_{8}$.

## 4. Fabrication and Evaluation of a Test Chip

**Figure 7:**
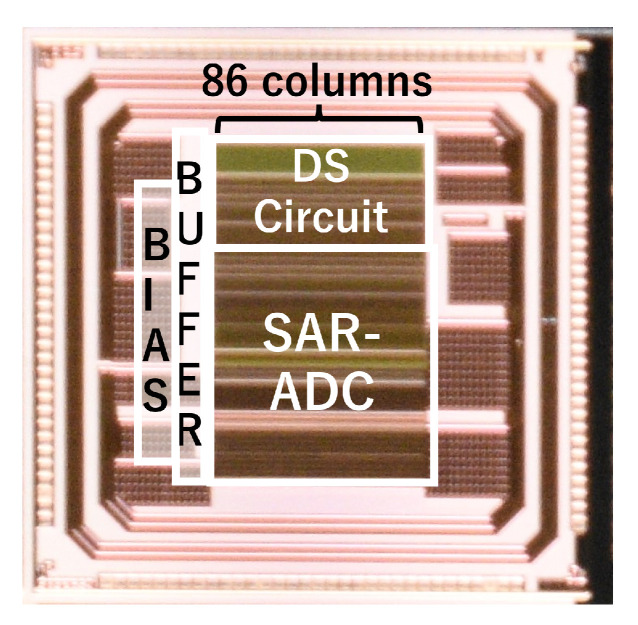
A photo of the fabricated test chip.

A test chip of the proposed readout circuit for the triple-gain LOFIC CIS was fabricated using a 0.18μm 1P5M CMOS process with MIM capacitors. A photo of the fabricated test chip is shown in Figure 7. The test chip contains the DS circuit, 10-bit SAR-ADC, BIAS, and BUFFER. The LOFIC pixel was not implemented in the test chip. There are 86 columns of DS circuits and ADCs with a pitch of 11.22μm laid out in parallel. The circuit area for the triple-gain LOFIC CIS increased by only 7.6% compared to the readout circuit for the dual-gain LOFIC CIS [25].

**Figure 8:**
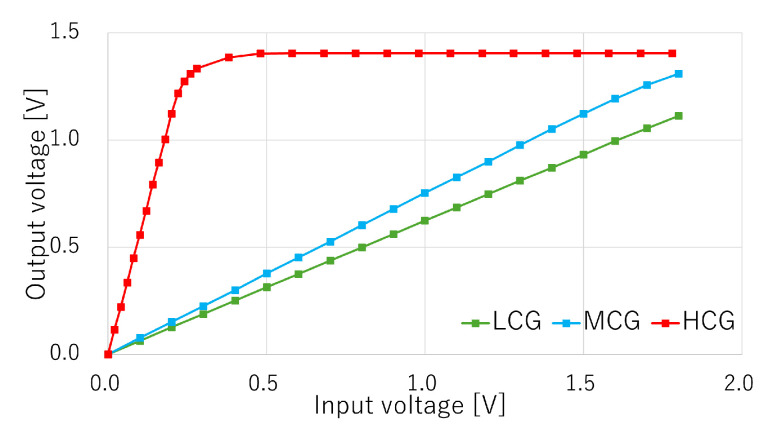
Measured input and output characteristics.

Figure 8 shows the measured input and output characteristics of the HCG, MCG, and LCG signals when the power supply voltage VDD is set to 2.8 V. The X-axis represents the voltage difference between the pixel reset level and signal level, and the Y-axis represents the output voltage swing referred from the ADC output. The measured circuit gains of the HCG and MCG signals were 5.67× and 0.76×, respectively. These values were lower than the target values of 8.0× and 0.84×, respectively. The root cause is suspected to be parasitic capacitance. The measured gain of the LCG signal was 0.62×, which was also lower than the target value of 0.72×. Table 1 summarizes the measured circuit gain and the estimated total gain. The estimated total gain is provided by $A_{cir}\times A_{pix}\times A_{SF}$, where $A_{cir}$ represents the measured circuit gains, such as 5.67 for HCG, 0.76 for MCG, or 0.62 for LCG; $A_{pix}$ represents the pixel conversion gains of $160\;\mu V/e^{-}$ for HCG and MCG and $10\;\mu V/e^{-}$ for LCG [16]; and $A_{SF}$ is the pixel SF gain of 0.85×, respectively.

The measured input-referred circuit noise is $0.28\;{\mathrm{mV}}_{\mathrm{rms}}$, $1.39\;{\mathrm{mV}}_{\mathrm{rms}}$, and $0.75\;{\mathrm{mV}}_{\mathrm{rms}}$ for the HCG, MCG, and LCG signals, respectively, provided by $N_{output}$/$A_{cir}$/$A_{SF}$, where $N_{output}$ is the measured output noise from readout circuit, $A_{cir}$ is the measured circuit gain, and $A_{SF}$ is pixel SF gain, respectively. The estimated input-referred noise is provided by $N_{m}$/$A_{pix}$, where $N_{m}$ is the measured input-referred circuit noise and $A_{pix}$ is pixel conversion gain [16], respectively. The input-referred circuit noise for the LOFIC pixel is also summarized in Table 2. For HCG, the input-referred circuit noise is minimized thanks to both high circuit gain and high pixel gain. Similarly, the input-referred circuit noise for MCG is lower than that for LCG thanks to its higher pixel gain even though the measured noise for MCG is greater than that for LCG because of the additional inverting attenuator.

**Figure 9:**
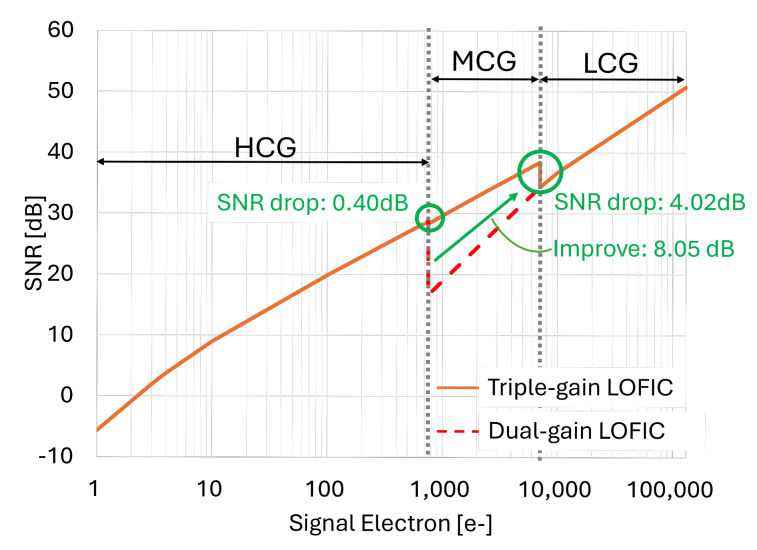
The SNR taking into account the optical shot noise, the pixel reset noise, and the measured noise of the proposed readout circuit.

Figure 9 shows the SNR, which takes into account the theoretical optical shot noise, the theoretical pixel reset noise, and the measured noise of the proposed readout circuit. It is noted that the 1/f noise generated by the pixel SF transistor is mitigated through double sampling for the HCG, MCG, and LCG signals.

At the switching point from HCG to MCG, the SNR drop is only 0.40 dB, which is very small, because the optical shot noise is dominant. The SNR drop at the switching point from MCG to LCG is 4.02 dB, but this will not be visible because the SNR exceeds 30 dB [27]. As the estimated SNR drop for the dual-gain LOFIC CIS is 12.07 dB, the proposed readout circuit improves the SNR drop by 8.05 dB for the triple-gain LOFIC CIS.

### Discussion

In the test chip design, 10-bit ADC was implemented. For HCG signal, the total conversion gain is $0.72\;e^{-}/\mathrm{LSB}$, which is based on the following parameters; a pixel conversion gain of $160\;\mu V/e^{-}$ [16], a circuit gain including a pixel SF gain of 6.8×, and ADC input window of 0.8 V for 1024 LSB. Therefore, the quantization noise of the readout circuit is less than one electron. Although a 12-bit ADC is preferable, a 10-bit ADC remains acceptable to meet the performance requirements of our target LOFIC CIS.

Also in the test chip design, the conversion period is constrained by the A/D conversion period, which is limited by the maximum clock frequency of the test chip. However, it is estimated that a 2 Mpixel triple-gain LOFIC CIS can achieve a frame rate of approximately 45 fps when used with a high-speed ADC originally designed for a 2 Mpixel 60 fps dual-conversion-gain CMOS image sensor [31].

Although the readout circuit for the LOFIC pixel has rarely been addressed in the published literature, we have provided a specification and performance comparison, as shown in Table 3. The readout circuit for a triple-gain LOFIC CIS reported in [24] employs a three-channel architecture. The single-channel readout circuit used in a global-shutter CMOS image sensor with in-pixel dual storage [32] can be applied to LOFIC CIS. However, since autozeroing of the comparator in the ADC is not feasible for the LCG signal, offset errors may lead to column fixed-pattern noise at the switching point to the LCG signal. The proposed triple-gain readout circuit improves the signal-to-noise ratio (SNR) by 8.05 dB, with no increase in pixel area, a 7.6% increase in circuit area, and a 25% reduction in frame rate compared to the case of a dual-gain readout circuit. The triple-gain LOFIC CIS incorporating the proposed readcout circuit will be suitable for cost-sensitive applications such as dashboard cameras and surveillance cameras.

## 5. Summary

We proposed an area-efficient readout circuit for the triple-gain LOFIC CIS in order to achieve a high SNR. The proposed readout circuit consists of an inverting amplifier, an inverting attenuator, a non-inverting attenuator, and an ADC. By utilizing amplifier and capacitor sharing techniques throughout the readout circuit, the area overhead of the readout circuit for the triple-gain LOFIC CIS is only 7.6% compared to that of the dual-gain LOFIC CIS. The SNR drops at the switching points from the HCG to MCG signals and from the MCG to LCG signals are 0.40 dB and 4.02 dB, respectively, representing an improvement of up to 8.05 dB. In future work, we will further evaluate the chip, including the LOFIC pixels.

## Figures and Tables

**Table 1:** Measured circuit gain and estimated total gain.

	HCG	MCG	LCG
Measured circuit gain [V/V]	5.67	0.76	0.62
Estimated total gain [$\mu V/e^{-}$]	771	103	5.27

**Table 2:** Measured and estimated input-referred noise of the proposed readout circuit.

	HCG	MCG	LCG
Measurement [${\mathrm{mV}}_{\mathrm{rms}}$]	0.28	1.39	0.75
Estimation [$e_{\mathrm{rms}}^{-}$]	1.75	8.69	75.5

**Table 3:** Performance comparison.

	[24]	[32]	This Work	
**Process**	180 nm 1P5M	90 nm 1P4M	180 nm 1P5M	
**Gain**	triple	dual	dual	triple
**#Readout circuit**	3	1	1	
**ADC resolution [bit]**	N/A	12	10	
**LCG autozeroing**	N/A	No	Possible	
**Readout period [μs]**	N/A	N/A	32	40
**Circuit area [$\mu m^{2}$]**	N/A	N/A	16,210	17,438
**SNR drop [dB]**	N/A	N/A	12.07	4.02

## Data Availability

The original contributions presented in this study are included in the article. Further inquiries can be directed to the corresponding author.
